# MRI visualization of maternal pelvic and perineal anatomical changes during the second stage of labor: a feasibility study

**DOI:** 10.1186/s41747-026-00757-4

**Published:** 2026-09-11

**Authors:** Jean-Christophe Maran, Olivier Ami, Tien Tuan Dao, Gérard Mage, Louis Boyer

**Affiliations:** 1 Univ. Lille, CNRS, Centrale Lille, UMR 9013-LaMcube - Laboratoire de Mécanique, Multiphysique, Multiéchelle, Villeneuve d’Ascq, Lille, France; 2 Ramsay Santé La Muette, Paris, France; 3 Université d’Auvergne, Clermont Ferrand, France; 4 Pôle Interhospitalier d’Imagerie Diagnostique et de Radiologie Interventionnelle, CHU, Clermont-Ferrand, France; 5 TGI, Institut Pascal, UMR 6602, UCA/CNRS/SIGMA, Aubière, France

**Keywords:** Feasibility study, Labor stage (second), Magnetic resonance imaging, Pelvic floor, Pregnancy

## Abstract

**Objective:**

Maternal pelvic anatomical changes during the second stage of labor (SSL) remain poorly understood because of the difficulty in visualizing internal structures *in vivo*. Mechanical models have attempted to predict these changes but lack real-time confirmation. This proof-of-concept study assessed the feasibility of using open-field magnetic resonance imaging (MRI) to visualize maternal and fetal structural movements during the SSL.

**Materials and methods:**

Twenty-seven term pregnant women were recruited for an open-field MRI study at a single center. Each participant was scheduled for scans in a 1-T open magnet within 2 h before labor onset and again during the SSL. Owing to logistical constraints, seven patients underwent both pre-labor and intrapartum MRI. Qualitative assessments of intrapartum pelvic anatomy changes were performed and supplemented by basic quantitative measurements of pelvic dimensions.

**Results:**

Intrapartum MRI was successfully performed in seven women (aged 23–34 years). All showed marked perineal expansion and deformation of the pelvic floor during fetal descent. The iliococcygeal portion of the levator ani flattened and reversed its curvature under fetal head pressure, corresponding to the perineal bulge. Posterior coccygeal deflection occurred in all the patients (mean coccyx–symphysis increase of ~ 0.8 cm), along with vaginal canal distension. Six patients had a partially filled bladder, which was displaced cranially as the fetal head descended. No adverse events occurred during MRI acquisition.

**Conclusion:**

Open-field MRI during the SSL is technically feasible and provides novel real-time observations of pelvic floor adaptations to fetal descent. These findings improve the understanding of intrapartum pelvic mechanics but require confirmation in larger cohorts.

**Relevance statement:**

Open MRI offers a way to observe directly how maternal pelvic structures adapt during childbirth in real time. This feasibility study’s insights are descriptive and hypothesis-generating, laying the groundwork for future research on protecting the pelvic floor during delivery rather than providing immediate clinical recommendations. By clarifying how maternal pelvic structures adapt to fetal descent, these MRI findings can inform strategies to minimize perineal trauma, guide interventions (*e.g*., episiotomy, not emptying the bladder before maternal efforts), and ultimately improve maternal postpartum pelvic floor health.

**Key Points:**

- Changes in the maternal pelvis during the second stage of labor remain poorly understood.
- Intrapartum open MRI can feasibly capture maternal pelvic anatomy in real time.
- Fetal descent markedly deforms the maternal pelvic floor (muscles, ligaments, and organs).
- Crowning is associated with significant anal sphincter stretching on MRI.
- Preliminary MRI observations may inform childbirth mechanics but are not yet generalizable.

**Graphical Abstract:**

**Figure:**
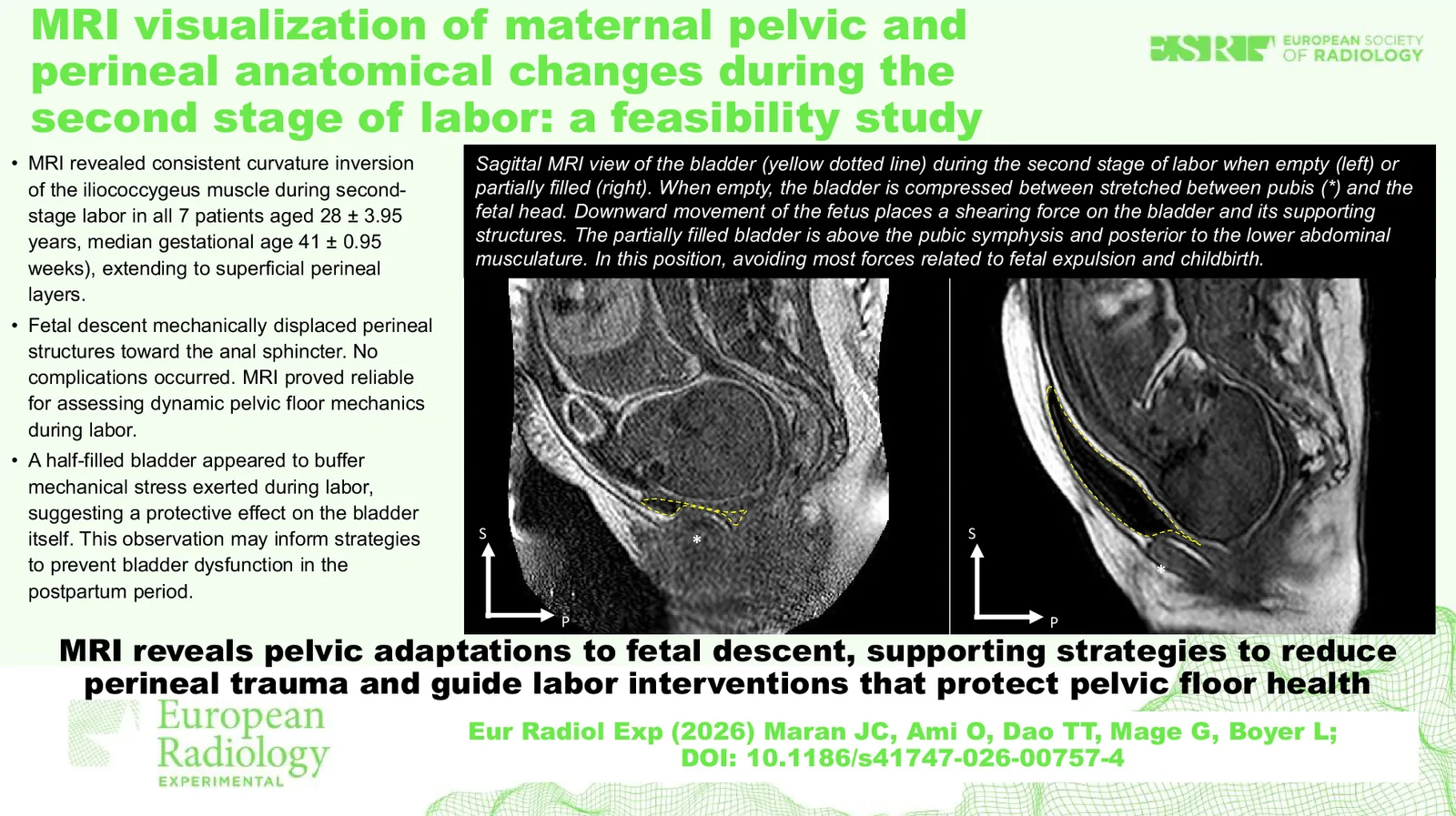

## Background

Mechanical and positional factors influencing labor progress are clinically important but remain difficult to investigate directly. Clinical examinations monitor fetal position and guide potential interventions but provide only an approximate assessment of maternal pelvic changes. The precise anatomical transformations that occur during intrapartum perineal stretching remain poorly characterized. Traditionally, digital vaginal examination was the sole intrapartum method used to assess the mother’s pelvis and fetal progression. This manual technique is limited to palpating accessible structures and is highly subjective and operator dependent, resulting in significant inaccuracies in determining fetal head position and station [1, 2]. Ultrasound has since improved the assessment of fetal head descent by visualizing the fetal skull relative to maternal bony landmarks, and its use has positively affected some clinical decisions [2, 3]. However, transperineal ultrasound cannot visualize certain deep pelvic structures; for example, the maternal ischial spines are obscured, and the flexion of the fetal head (fetal attitude) or the state of the maternal soft tissues cannot be fully evaluated with ultrasound because of bony interference and the fetus itself [4]. Real-time imaging of maternal pelvic floor structures during the second stage of labor (SSL) has remained elusive with conventional modalities.

Open-field magnetic resonance imaging (MRI) offers a potential solution by providing clear visualization of internal pelvic anatomy without ionizing radiation [5–7]. MRI can depict both bony and soft-tissue structures in multiple planes with high contrast, making it well-suited for examining the maternal pelvis and fetal position concurrently. Following the first report of real-time MRI during childbirth by Bamberg et al in 2012, we sought to further explore this imaging approach [6]. We conducted an initial exploratory study using an open 1-T MRI system to perform anatomical evaluations of maternal pelvic and perineal structures in a cohort of laboring patients. Scans were obtained shortly before the onset of labor and then again during the SSL in the same patients, allowing paired comparisons of pre-labor and intrapartum anatomy in the axial, sagittal, and coronal planes.

This study was designed as a feasibility demonstration and descriptive analysis of intrapartum MRI. We aimed to demonstrate that open MRI can safely capture maternal pelvic anatomy during active labor and to describe the key movements and deformations of pelvic structures during the SSL. The focus was on visualizing anatomical changes associated with fetal descent in real time rather than on drawing generalizable clinical conclusions.

## Methods

### Study design and setting

This investigation was a single-center prospective pilot study intended to establish the feasibility of intrapartum MRI. The research protocol (IMAGINAITRE study) received approval from the local Institutional Review Board (Comité de Protection des Personnes Ile-de-France II, approval #IDRCB2012-A01469-34) and authorization from the French National Agency for Drug and Medical Product Safety‒ANSM. All the participants provided informed consent. The study was conducted at a maternity center equipped with an adjacent MRI suite, a setup crucial for minimizing transport time and ensuring patient safety during labor imaging.

### Participants

Healthy pregnant women aged 23–34 years with singleton pregnancies at term (≥ 39 weeks’ gestation) were enrolled. Both primiparous and multiparous patients (up to two prior deliveries) were included. Key inclusion criteria included cephalic presentation and an absence of known factors that might complicate labor or delivery. The exclusion criteria included any condition posing maternal or fetal risk or precluding MRI (*e.g*., non-cephalic presentation, prior uterine scar, multiple gestation, urgent obstetric indications, abnormal fetal heart tracing requiring imminent intervention, contraindications to MRI, or inability to provide consent). Twenty-seven patients met the selection criteria and were included (Table 1). Of these, seven women ultimately underwent the full imaging protocol with MRI scans both before labor onset and during the SSL. The remaining 20 enrolled patients had only the pre-labor MRI scan, as they did not complete the intrapartum scan (primarily because of the unpredictable timing of labor progression and logistical limitations in coordinating immediate MRI during active labor).

**Table 1:** Flow diagram and MRI sequences parameters

Parameter	3D T1-weighted (E-THRIVE)	Dynamic sagittal T1 (DYN_BFFE)
Field strength	1.0 Tesla (open MRI, Panorama, Philips Healthcare)	1.0 Tesla (open MRI, Panorama, Philips Healthcare)
Sequence type	3D gradient echo, continuous slices	Dynamic single-shot sequence
Fat suppression	None (no FAT-SAT)	None (no FAT-SAT)
TE (ms)	1.62 ms	1.65 ms
TR (ms)	3.2 ms	3.29 ms
Flip angle (°)	10°	45°
Matrix	448 × 448	448 × 448
TFE factor	82	82
NSA	1	1
Number of slices/dynamics	160 overlapping slices	100 overlapping slices
Acquisition voxel size	2.2 mm	1.17 mm
Reconstructed voxel size	1 mm	1 mm
Acquisition time	~ 30 s	~ 1 min (dynamic)

### Imaging procedure

Each patient who completed the study received two MRI examinations: one within approximately 2 h prior to the onset of labor (at very late term, before regular contractions and pushing began) and a second scan during the SSL, just before the start of expulsive efforts (Table 2). Entry into the SSL was defined clinically by full cervical dilation combined with fetal head engagement (the head descending past the plane of the superior pelvic strait on clinical examination). All intrapartum imaging was performed with the patient in a modified supine position on the MRI table, closely mimicking the standard delivery position, to maintain consistency with the pre-labor scan positioning. The MRI unit used here was a Philips Panorama 1-T open-configuration scanner (Philips Healthcare) located in the same building as the labor ward. This proximity allowed for the rapid transfer of patients between the labor room and the MRI suite. The transport time from the maternity ward to the MRI and back was less than 3 min each way in all the patients, ensuring minimal interruption of obstetric care. Throughout the MRI sessions, full labor and fetal monitoring capabilities were in place, and obstetrical and radiology teams worked in tandem to maintain patient safety.

**Table 2:** Characteristics of the 7 study patients

Patients	Age (years)	Parity	Fetal age (weeks)	MAGNIN score (cm)	Fetal weight (g)	Height (cm)	Perineum	Delivery type	Descent according to DIETZ (mm)	Angle according to DIETZ (degrees)
Patient 1	23	1	40	25.1	3,380	51	Intact	Normal	27.37	21.6
Patient 2	28	2	41	26.7	4,000	53	Scuffing	Normal	23.07	17.6
Patient 3	28	1	41	26.1	3,755	55	Intact	C-section at full dilation	31.15	26.5
Patient 4	31	2	41	25.1	4,525	54	Single tear	Normal	53.79	48.5
Patient 5	31	2	39	27.5	3,095	49	Intact	Normal	30.56	24.2
Patient 6	34	3	39	27.1	4,145	52	Single tear	Normal	29.91	26.1
Patient 7	24	1	41	25.6	3,625	51	Intact	C-section at full dilation Failed forceps	25.54	25.3
Median	28	2	41	26.1	3,755	52	…	…	29.91	25.3

### Safety considerations

Obstetric and neonatal care teams were present during all MRI acquisitions to monitor maternal and fetal well-being continuously. Standard labor monitoring, including intermittent fetal heart rate surveillance, was maintained as much as possible. A portable monitor by AN24 (Monica Healthcare) was used and disconnected intermittently during the acquisition period, which was always less than 10 min. Because fetal heart rate could not be monitored inside the scanner, monitoring was interrupted during MRI; cumulative interruption time did not exceed 10 min per patient. Importantly, at no point were maternal or fetal safety compromised; patients received the same level of vigilance and care as they would in the delivery room, with emergency interventions available immediately if needed. No adverse events occurred as a result of the MRI procedure.

### MRI protocol

The same imaging protocol was applied to both the pre-labor and intrapartum scans for consistency. Each MRI examination consisted of several rapid sequences without intravenous contrast. Key sequences included (1) multiplanar T1-weighted turbo field echo acquisitions for anatomical overview; (2) a three-dimensional (3D) “enhanced-T1‑weighted high‑resolution isotropic volume examination”—e-THRIVE sequence focusing on bony anatomy and fetal head position allowing for potential reconstruction; and (3) a dynamic, T1-weighted sequence acquired in the mid-sagittal plane centered on the birth canal, lasting approximately 60 s, to capture fetal head movement and pelvic floor deformation in real time (Table 1). Each individual sequence was fast (on the order of 20–30 s for static images and ~ 60 s for the dynamic run), and the total imaging time for the intrapartum study averaged 8 min (with no single continuous scanning period exceeding approximately 2 min). These short acquisition times were chosen to accommodate maternal movement between contractions and to minimize any delay in delivery.

All the images were acquired with a field of view covering the maternal pelvic region and fetal head. Immediately after imaging, the patients were transported back to the labor room or operating room for delivery, if not already delivered in the MRI. In two cases in which emergent Cesarean delivery became necessary following the intrapartum MRI, the transfer time from the MRI suite to the operating theater was approximately 2.5 min, demonstrating that the MRI protocol was compatible with urgent obstetric management.

### Image analysis

MRI data from before and during labor were compared to evaluate anatomical changes. To quantify fetal head descent on MRI, we adopted the method described by Dietz et al for ultrasound and adapted by Bamberg et al for MRI [8, 9]. Specifically, on mid-sagittal images, we defined a reference line perpendicular to the long axis of the pubic symphysis at its inferior margin. We then measured the vertical distance from this reference line to the lowest point of the fetal skull on the pre-labor scan and on the intrapartum scan, with the difference representing the descent of the fetal head during the second stage. We also measured the angle of progression of the fetal head (the angle between the line through the long axis of the symphysis and a line connecting the inferior pubis to the leading point of the fetal head) at both time points.

To assess bony pelvic movement and perineal expansion, we measured two anteroposterior pelvic dimensions on both scans: the distance from the inferior edge of the pubic symphysis to the coccyx (SCSP) and the distance from the coccyx to the sacral promontory (CP). Changes in these distances from pre-labor to intrapartum images were used to detect any posterior or anterior shift of the pelvic outlet. We also calculated the angle between the SCSP and CP lines (the “interdiameter angle”) as an indicator of pelvic outlet widening or narrowing. Additionally, to quantify perineal widening, we measured the angle subtended at the perineal body (central tendon) by lines drawn to the inferior pubis and to the coccyx and compared this “perineal expansion angle” before and during labor.

3D reconstructions (additional analysis): For a subset of anatomical structures, we performed three-dimensional reconstructions to visualize spatial displacements. In particular, the tendinous arch of the levator ani (TALA) on each side was segmented on 3D MRI sequences acquired before and during labor. Using 3D Slicer software (version 5.0.1; open-source), the pre-labor and intrapartum pelvic models were co-registered by aligning bony landmarks (*e.g*., the pelvic brim, sacrum, and acetabula) as fixed reference points. This approach allowed for direct comparisons of the position of the TALA between time points. Seven anteroposterior measurements along each TALA were taken to quantify its downward displacement (caudal shift) due to labor. All segmentation and measurements were performed by a trained operator and verified by a senior radiologist for consistency.

## Results

### Patients

Seven of the 27 enrolled patients achieved full dilation and underwent intrapartum MRI during the SSL. These women were between 23 and 34 years old (mean age 28 ± 3.9 years). Three patients were primiparous, three were secundiparous, and one was tertiparous. All seven had reached at least 39 weeks of gestation at the time of labor (four were beyond 40 weeks). All participants had a Magnin pelvic score [10] > 25 (median 26.1, range 25.1–27.5), indicating adequate bony pelvic dimensions; none of the patients had an anatomically small pelvis according to this scoring.

All seven entered spontaneous labor and were fully dilated at the time of MRI. Five had uncomplicated spontaneous vaginal deliveries. The remaining two underwent unplanned Cesarean deliveries for arrest of descent at full dilation (including one after failed forceps). Both occurred in post-term pregnancies. No shoulder dystocia or clinically significant cephalopelvic disproportion occurred. The neonatal birth weights ranged from 3,095 g to 4,525 g (median 3,755 g). No patients experienced postpartum hemorrhage, and the Apgar scores were reassuring. These limited outcomes cannot support statistical comparison but indicate that imaging did not adversely affect delivery.

### Imaging logistics

Among the 27 women who were recruited, only seven ultimately underwent a second MRI during active labor (Tables 2 and 3). This low yield was primarily due to logistical challenges and the unpredictable timing of labor. Coordinating transfer to the MRI suite during the narrow window of second-stage labor was difficult for many patients (*e.g*., rapid labor progression or clinical contraindications to transport). The use of an open-configuration MRI system was essential, as conventional closed-bore units do not permit the semi-upright positioning, access for maternal monitoring, or team proximity required during active labor [7]. Despite this limitation, our protocol demonstrated that MRI scans during labor are achievable under carefully managed conditions. An open MRI system and a co-located maternity ward were critical for imaging these patients without compromising maternal-fetal safety.

**Table 3:** Fetal head position along the birth channel

Patients	Presentation at upper brim	Presentation at middle brim	Presentation at lower brim
Patient 1	LOA		OA
Patient 2	LOA	OA	OA
Patient 3	LOA	LOA	
Patient 4	LOA	LOA	OA
Patient 5	LOA	OA	OA
Patient 6	LOA	OA	OA
Patient 7	OP	OP	OA
Median	28	2	41

All fetuses presented themselves in a left LOA position, except one in an OP position. Except for one missing entry, all fetuses rotated at the middle brim to present their own OA position to the lower brim

*LOA* Left occiput anterior, *OA* Occiput anterior, *OP* Occiput posterior

With respect to the seven successful cases, pre-labor MRI was performed within 12 h of delivery in five patients (5 days before one and 16 days before another because of scheduling constraints). Intrapartum MRI was performed during second-stage labor at a median of ~ 32.5 min (range 14–57 min) before the actual time of birth. On average, the intrapartum MRI took 8 min, with no single scan exceeding 10 min. Transfer from the labor room to the MRI suite took approximately 2.5 min (median 2:36 min:s, range 2:24–3:13 min:s). In the two cases that required emergency Cesareans, transfer back to the operating room also took approximately 2.5 min (range 2:27–3:35 min:s). These intervals show that the MRI introduced only a brief interruption in the obstetric workflow. These findings support the technical feasibility of real-time pelvic imaging during labor, with minimal disruption when appropriate infrastructure and coordination are in place.

### MRI findings during the SSL

#### Pelvic floor deformation

**Fig. 1:**
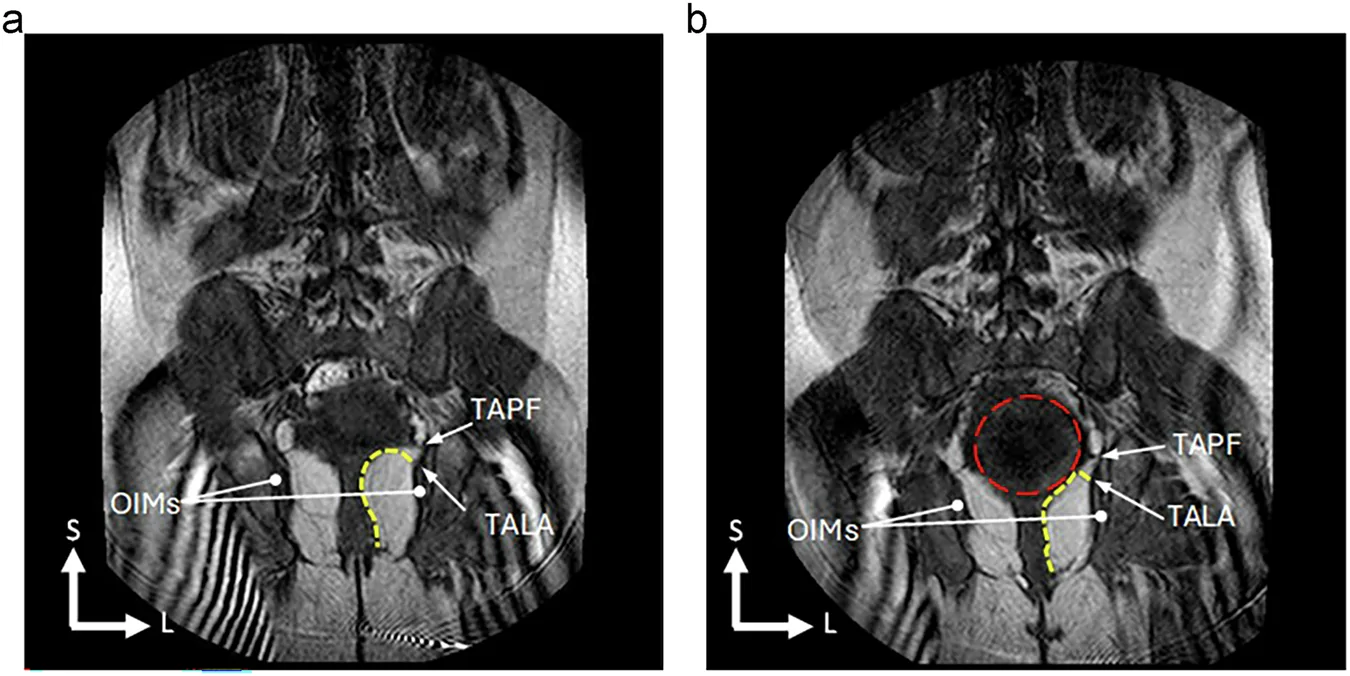
Coronal MRI highlighting the reversal of curvature of the iliococcygeal muscles (yellow dotted line) during labor (**b**). Left iliococcygeus convex side up before labor (**a**). Iliococcygeal concave side up after mobile fetal engagement marked with a red dotted line (**b**). Insertions of the TALA and the TAPF are highlighted (arrows). MRI, Magnetic resonance imaging; TALA, Tendinous arch of the levator ani; TAPF, Tendinous arch of the pelvic fascia

**Fig. 2:**
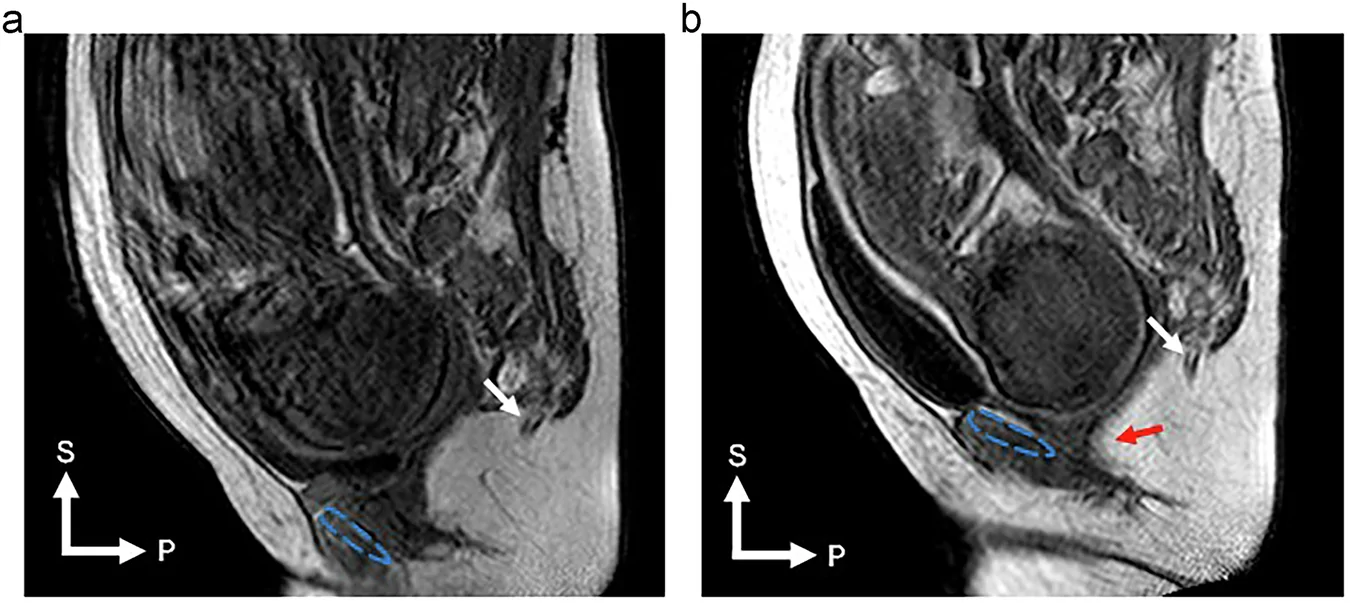
Sagittal MRI before (**a**), during the SSL (**b**). Fatty tissue (red arrow) between the vagina (not visible) and the sacrotuberous ligament (white arrows) shifted to the paramedian sagittal plane in all 7 patients during the SSL. The local fat moved to fill zones of weaker resistance inferiorly and para-medially, with the greatest movement being observed in the region of the anal sphincter. The net result of these changes was perineal bulging. The blue dotted line traces the pubic symphysis. MRI, Magnetic resonance imaging; SSL, Second stage of labor

In all seven patients, the descent of the fetal head into the pelvis produced clinically observed changes in the maternal pelvic floor anatomy. The most striking finding was the compression and flattening of the LAM, particularly the iliococcygeus, as the fetal head was engaged. Before labor, the iliococcygeus formed a convex hammock supporting the pelvic organs; during second-stage labor, it became compressed and inverted into a concave shape (Figs. 1 and 2). This deformation was accompanied by the widening of the urogenital hiatus and stretching of the puborectal fascia. Downward fetal head pressure displaced adjacent connective tissue and fat posteriorly, producing the classic perineal bulge. All seven women exhibited significant perineal expansion on MRI corresponding to the clinical distension of the perineum as the head crowned.

Consistently, we observed a posterior shift in bony and ligamentous structures with fetal descent. The coccyx was pushed backward in every case. Quantitatively, the SCSP increased by an average of 0.78 ± 0.41 cm (range 0.48–1.35 cm), and the distance from the coccyx to the CP increased by an average of 0.80 ± 0.30 cm (range 0.76–1.25 cm). This change reflected the nutation of the sacrococcygeal joint, increasing the anteroposterior diameter of the pelvic outlet. Despite these shifts, no lateral widening of the bony pelvis occurred at the inlet, mid-pelvis, or outlet. The pelvic architecture remained intact, and birth canal expansion occurred primarily through soft tissues and in the anteroposterior dimension. The ‘interdiameter angle’ between the SCSP and CP decreased by 5.3° ± 2.7° (range 2.8–9.3°) from pre-labor to the SSL, which is consistent with the sacral and coccygeal tilt accommodating the fetal head. On the intrapartum images, the fetal head markedly distended the vagina during its descent.

#### Bladder displacement

**Fig. 3:**
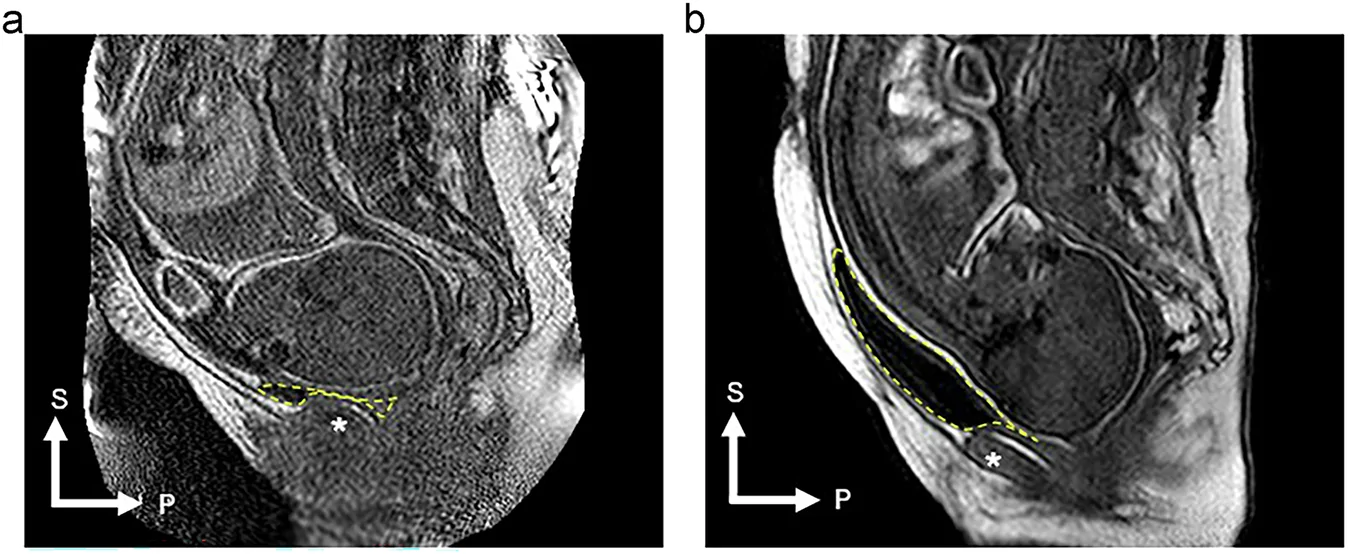
Sagittal MRI of the bladder (yellow dotted line) during the SSL when empty (**a**) or partially filled (**b**). When it is empty, the bladder is compressed between the pubis (*) and the fetal head. The downward movement of the fetus places a shearing force on the bladder and its supporting structures. The partially filled bladder is above the pubic symphysis and posterior to the lower abdominal musculature. In this position, it appears to avoid most of the forces related to fetal expulsion and childbirth. MRI, Magnetic resonance imaging; SSL, Second stage of labor

Six of the seven patients had a partially filled urinary bladder visible on their intrapartum MRI (the remaining patient’s bladder was essentially empty at the time of imaging). In those with some bladder volume, the organ was clearly displaced upward, rising above the pubic symphysis as the fetal head moved down into the pelvis. In the single patient with an empty bladder, the walls appeared flattened and pulled inferiorly by the descending head, suggesting direct compression (Fig. 3). A partially filled bladder did not impede fetal head descent; the head engaged normally, displacing the bladder out of the way. The angles of progression and descent distances were similar between filled and empty bladder patients. This finding is intriguing given standard practice, but the small sample precludes definitive conclusions. No complications, such as bladder injury or retention, were noted.

#### Cervical and perineal soft tissue changes

**Fig. 4:**
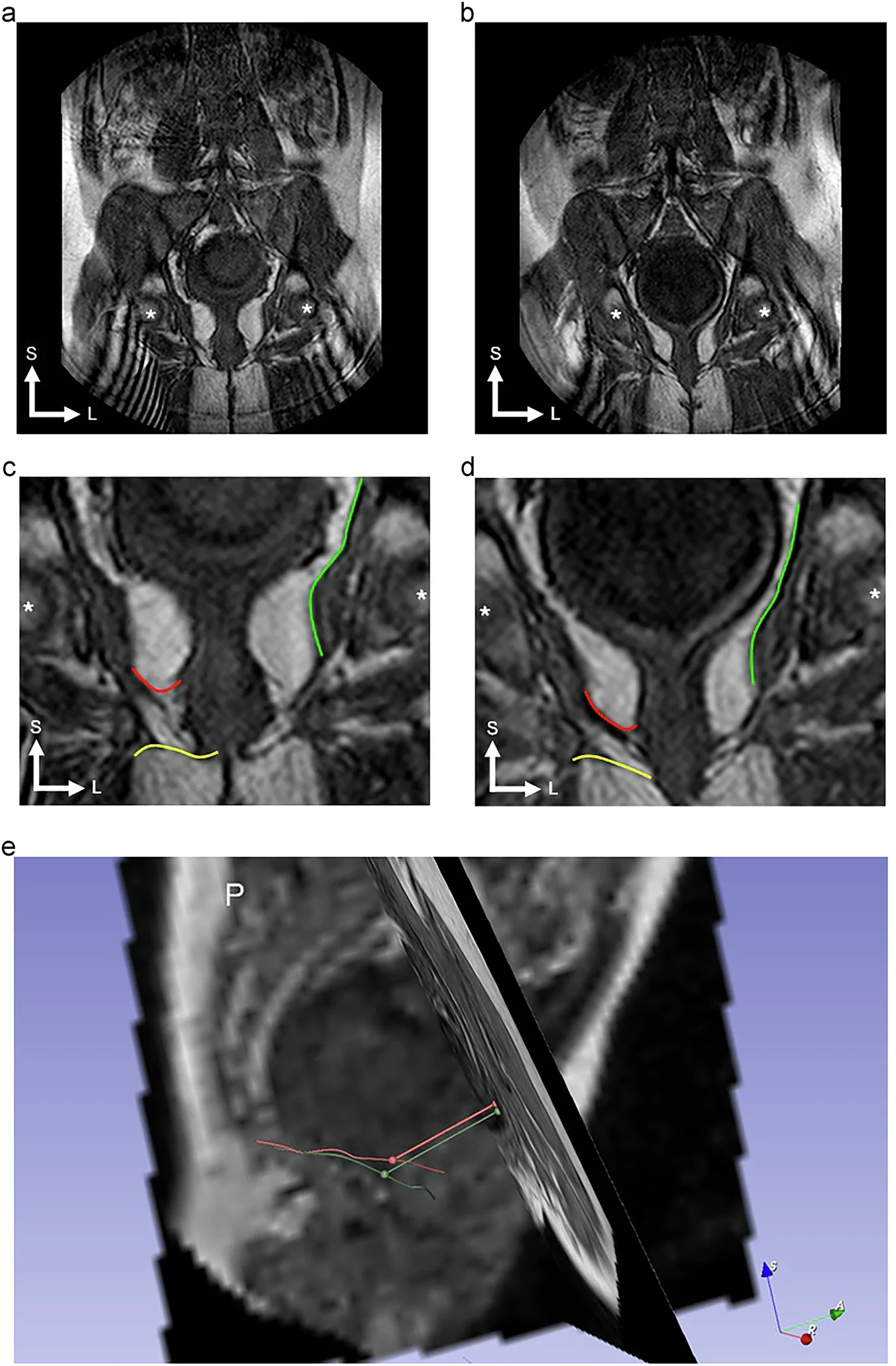
MRI of the pelvis in the coronal plane before entering labor (**a**, **c**) and during the SSL (**b**, **d**). The OIM (green line) was pushed externally by the descending fetal head. The superficial (yellow line) and deep (red line) transverse muscles were also flattened, compressed together, and shifted inferiorly, as indicated by the change in distance from the femoral head (*). A 3D view comparing the TALA before (red 3D line) and during (green 3D line) the SSL (**e**) based on image registration. The orthogonal distance between the TALAs and the upper brim plane decreases during the SSL. 3D, Three-dimensional; MRI, Magnetic resonance imaging; SSL, Second stage of labor; TALA, Tendinous arch of levator ani

On all the intrapartum scans, the cervix was fully dilated and effaced, no longer forming a distinct canal around the fetal head. The thinning of the cervical myometrium corresponded to the fetal head entering the mid-pelvis. As the head passed the levator ani muscle (LAM) level, the muscles and overlying perineal fat and connective tissues were pushed outward and downward (Fig. 4). Expansion of the superficial perineal tissues began as soon as the fetal head contacted the LAM plane. Lateral displacement was limited by the sacrotuberous ligaments, which acted like ‘guide rails’ and limited lateral bulging. Thus, distension was directed primarily posteriorly toward the coccyx and anus.

**Fig. 5:**
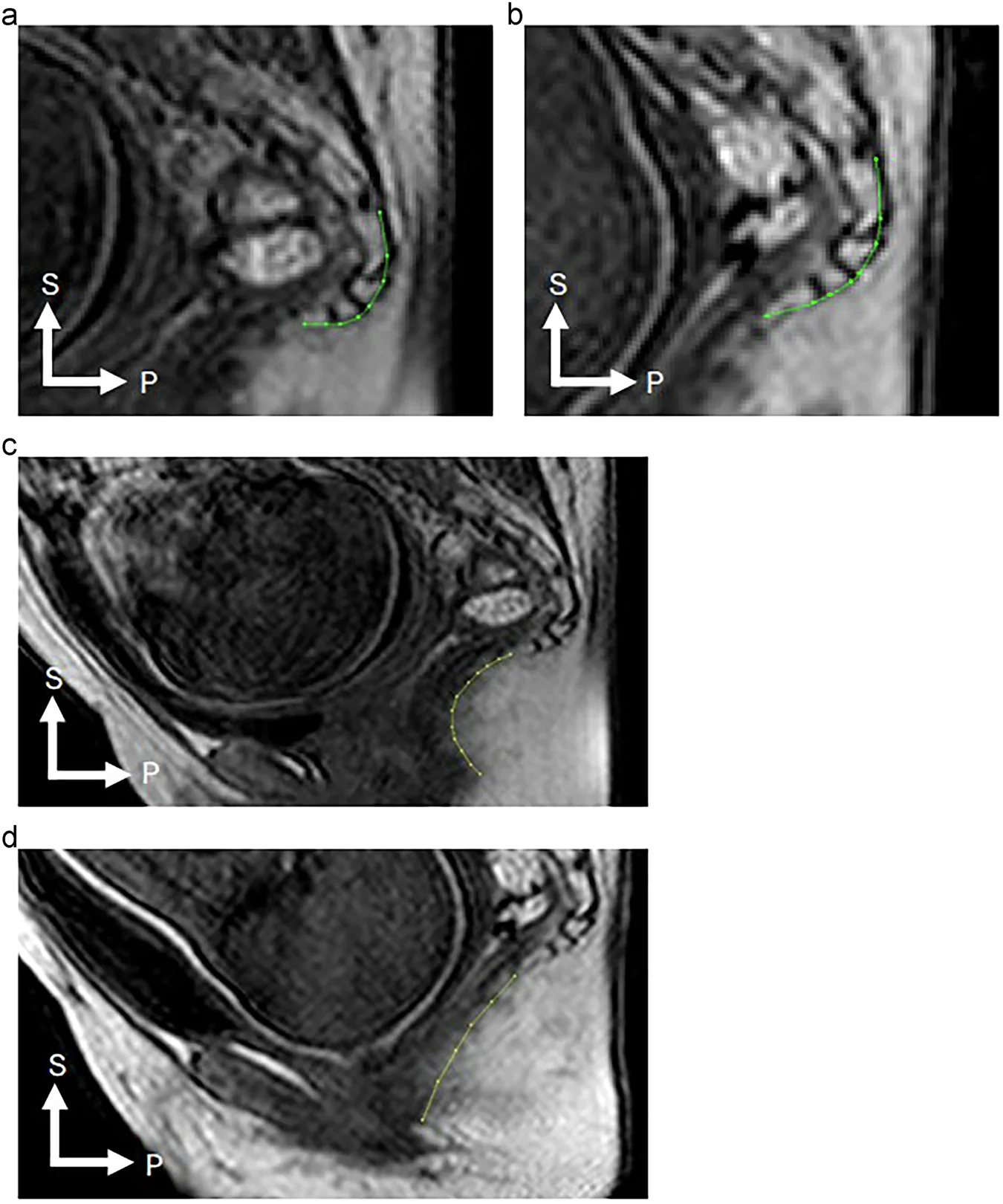
Sagittal MRI before the onset of labor (**a**, **c**) and during the SSL (**b**, **d**), patient #5 close-up of the coccyx before labor (**a**), which is concave anteriorly. During the SSL (**b**), the head is at the middle brim in the OA position, and the coccyx shows posterior nutation. The perineal raphe (in yellow) before the onset of labor is horizontal (**c**). During the SSL (**d**), the perineal raphe was pushed outward, resulting in a more linear appearance. MRI, Magnetic resonance imaging; OA, Occiput anterior; SSL, Second stage of labor

Deep pelvic connective tissues were also visualized. Paravaginal and pararectal connective tissue-fatty areas, components of the sacrorectogenitopubic lamina, beneath the LAM (Figs. 2 and 4), shifted inferiorly and posteriorly as the head engaged and descended. This downward displacement of the pelvic connective tissue dragged the attached deep and superficial transverse perineal muscles with it, contributing to a crowding of the muscular structures toward the midline (Fig. 5).

#### Anal sphincter and pelvic floor support

**Fig. 6:**
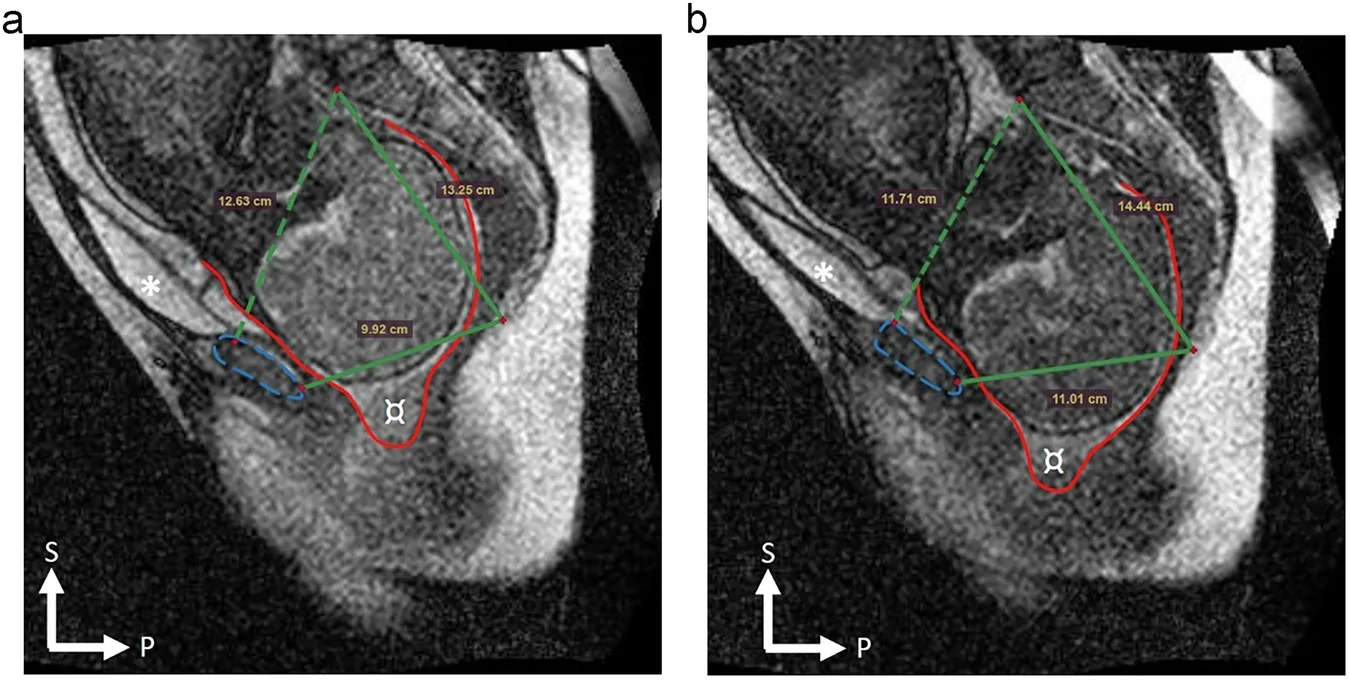
Dynamic sagittal MRI. The fetus (red line) presents himself in an OA position at rest (**a**) and with maternal expulsive efforts (**b**). The descent of the fetal head compared to the upper brim plane (green dotted line) is visible. The pubis (blue dotted line) serves as a pivot point for the de-rotation of the fetal head. A subcutaneous hematoma is visible (¤). The hematoma follows the de-rotation of the head around the pubis during expulsive efforts. A partially filled bladder is seen above the level of the pubic bone (*). MRI, Magnetic resonance imaging; OA, Occiput anterior

The greatest degree of soft tissue movement was observed in the region of the anal sphincter complex. As the fetal head crowned, significant stretching of the anal sphincter complex was observed by MRI in all the cases. Head engagement caused marked posterior deflection of the perineal body and anal sphincter. The midline raphe was pulled posteriorly, rotating from a horizontal to a more vertical orientation during crowning (Fig. 6). The lateral puborectalis fibers were forced apart during maximum fetal head distension. These changes were accompanied by the dilation of the anal canal. In three patients, anal sphincter measurements revealed length and width increases of 1.0 ± 0.6 cm and 0.9 ± 1.1 cm (range -0.08 to 2.08 cm), respectively, from pre-labor to crowning. When expressed as proportional deformation, the longitudinal dimension of the anal sphincter increased by a mean of 27.9% (range 10.7 to 47.0%). Transverse deformation showed wider dispersion, with a mean increase of 31.3% (range -5.8 to 64.9%), reflecting widening in two cases and localized thinning in one. In the remaining four patients, quantification was not possible, but the qualitative patterns were similar. In the limited data available, greater fetal head descent correlated with a smaller residual sphincter width, suggesting more pronounced thinning with higher pressure. This inverse relationship was noted, although the small sample precluded formal statistical analysis.

#### Tendinous structures

Coronal MRI consistently revealed that the TALA was inserted into the medial fascia of the obturator internus muscle and provided attachment to the lateral aspect of the iliococcygeal component of the LAM. The tendinous arch of the pelvic fascia appeared as the superior fascial layer overlying the iliococcygeus origin. Both structures demonstrated measurable inferior and posterior displacement during fetal head descent, reflecting dynamic loading of the pelvic fascial support system. The magnitude and spatial orientation of TALA displacement varied along its anteroposterior course, with the most pronounced excursions occurring in the anterior segment and progressively smaller movements occurring medially and posteriorly (Table 4). Minimal variation was observed in the internal distance between the left and right TALAs, indicating that deformation occurred predominantly in the sagittal plane. Surrounding adipose tissues were consistently displaced outward and caudally as the fetal head advanced, illustrating a global redistribution of perineal and paravaginal soft tissues under mechanical loading.

**Table 4:** Quantitative displacement of the tendinous arch of the levator ani (TALA) during fetal head descent on coronal MRI

Anatomical segment	Side	Mean displacement (mm)	SD (mm)	Range (mm)
Anterior trajectory	Right	12.6	7.6	1.5–21.0
	Left	16.2	6.8	5.2–25.2
Medial trajectory	Right	9.1	5.0	1.5–17.8
	Left	12.0	4.9	3.7–19.8
Posterior trajectory	Right	4.1	3.5	-1.6 to 10.2
	Left	8.8	6.5	-1.1 to 21.2
Inter-TALA distance	Bilateral	0.5	1.3	-2.0 to 2.8

## Discussion

Before real-time imaging, understanding of the functional birth canal anatomy relied on cadaver studies, non-labor imaging, and gross clinical observation [11, 12]. While the static anatomy of the female pelvis and pelvic floor is well documented, the dynamic changes associated with childbirth remain poorly described. This study revealed that open MRI can directly visualize these dynamic changes during labor. MRI performed shortly before labor and during the SSL in seven patients provided real-time insight into maternal ligament, muscle, and organ movement. This imaging approach revealed childbirth mechanics with details not achievable by physical examination or ultrasound [3]. Digital examination, while historically central to intrapartum assessment, is known to suffer from limited reproducibility and operator dependence, especially for estimating fetal head station and position [1, 2]. MRI clearly delineated fetal head–pelvis relationships, contributing to a better understanding of how fetal descent is accommodated by maternal tissues.

Our findings in these seven cases were consistent in several aspects. First, we observed a characteristic deformation of the LAM group (specifically the iliococcygeal component) as the fetal head was engaged. The LAM, which was normally dome-shaped, was flattened and even reversed by fetal head pressure. This deformation enlarged the opening of the pelvic floor and resulted in posterior perineal bulging. Concomitantly, the surrounding fascia and fat were displaced, contributing to the visible perineal bulge. Downward forces shifted the median raphe posteriorly and inferiorly and displaced the perineal body forward and downward. Together, these movements resulted in substantial stretching of the anal sphincter ring. The rectum, lying within this complex, was notably compressed—its walls thinned, and its contents (air or stool) were expelled as the head pressed through. This set of observations aligns with clinical phenomena: obstetricians often note that as the head crowns, the anus dilates and sometimes the stool is evacuated. The extreme stress imposed on the anal sphincter and pelvic floor muscles during this process might contribute to the high rates of postpartum pelvic floor disorders, such as anal incontinence and dyspareunia, as reported in the literature [13–16]. Our imaging provides visual evidence of the anatomical basis for these injuries. The degree of anal distension on MRI matched the delivery-room observations, suggesting that the imaging reflects true intrapartum mechanics.

Although rotational changes and molding of the fetal head were visible on MRI, these features were not analyzed quantitatively in this feasibility study and were therefore not included in the results section to avoid overinterpretation.

We also documented changes during maternal pushing that clarified pelvic mechanics. With each push, the coccyx underwent nutation, swinging anteriorly relative to the sacrum. This motion increased the anteroposterior pelvic outlet diameter, as reflected by the increase in the CP on MRI. No gapping or separation of the pelvic bones was observed; the pelvis behaved as a rigid ring, and the expansion of the birth canal was achieved by the combined effect of the sacrococcygeal nutation and the extreme distension of soft tissues. The sacral and coccygeal tilt helped maintain the anteroposterior dimensions despite pelvic floor expansion and thinning. This observation suggests that the bony pelvis provides limited accommodation during delivery beyond slight coccygeal movement; thus, space is created primarily by soft tissues. Conservation of the pelvic outlet diameter *via* bony mechanics likely complements soft tissue stretching to facilitate delivery.

These MRI-based measurements are consistent with prior transperineal ultrasound findings regarding fetal head engagement and the angle of progression, particularly those reported by Dietz and Bamberg [8, 9], and extend them by providing direct visualization of associated soft-tissue deformation.

Notably, the behavior of the maternal bladder during the SSL was observed. In six of the seven patients, the partially filled bladder was displaced upward above the fetal head as labor progressed. Thus, the bladder was lifted out of the pelvic inlet area, seemingly protecting it from direct compression (Fig. 3). In the single empty bladder case, the bladder lay collapsed in the pelvis and was directly subjected to shearing forces. This distinction may have clinical implications. It is common for obstetric caregivers to empty the bladder at full dilation, under the assumption that an empty bladder allows more room for the fetus to descend and reduces obstruction. Our preliminary data suggest that a partially filled bladder does not impede descent and may even assume a protective position. Conversely, an emptied bladder may fall into the pelvis and be more exposed to trauma from the fetal head. These findings raise the hypothesis that complete emptying before pushing could remove a protective “cushion” and increase compression risk. However, we emphasize that this insight is based on a very small number of cases. While thought-provoking, it is not grounds for changing clinical practice at this time. Further studies with larger populations are needed to evaluate the impact of bladder volume on labor outcomes and pelvic organ integrity. Until then, the protective effect of partial bladder filling remains a hypothesis generated by this proof-of-concept study.

There are several important limitations to our study. The primary limitation is the small sample size (*N* = 7), which was dictated by the practical challenges of performing MRI during labor. With so few observations, the findings are descriptive and exploratory. We did not attempt formal statistical analysis, as the study was not powered for hypothesis testing. As a result, our conclusions are hypothesis-generating rather than confirmatory. Second, the MRI captured only a brief interval of the SSL. Scan timing was opportunistic, allowing the observation of only part of the dynamic delivery process. Additional or more extreme changes at the time of delivery may not have been captured. Third, we lacked a comparative modality (*e.g*., ultrasound) to correlate with the MRI findings. Thus, some measurements cannot be validated against standard clinical assessment tools. Lastly, this study required a highly controlled setting and substantial resources. An open MRI adjacent to the labor ward was critical; such infrastructure is rare and may limit replicability. A multidisciplinary team (obstetrics, anesthesiology, radiology, and neonatology) was required to ensure maternal and fetal safety. Attention to positioning, comfort, and motion minimization (breathing, contractions) was essential for diagnostic images. These conditions underscore that intrapartum MRI is technically challenging and that success hinges on meticulous planning and teamwork.

Despite these limitations, our experience provides valuable technical insights. We showed that MRI can be performed during active labor without significantly disrupting clinical care. High-resolution imaging of maternal and fetal anatomy during childbirth opens new research avenues. This study demonstrates feasibility and provides meaningful information about childbirth mechanics. These insights deepen the understanding of anatomy and may help generate hypotheses for preventing pelvic floor injury. Future studies could involve multiple centers or integrate MRI with clinical outcomes to expand these findings. Although limited, these findings demonstrate the feasibility of intrapartum MRI and highlight its potential to explore childbirth mechanics.

In conclusion, this study revealed that performing open-field MRI during the SSL is technically feasible and safe in a controlled setting. MRI-based observations provided real-time visualization of how the maternal pelvic anatomy accommodates the descending fetus, offering novel descriptive insights into the mechanics of childbirth. Key anatomical changes—such as LAM flattening, coccygeal movement, and perineal stretching—were directly observed, contributing to the qualitative understanding of labor physiology. However, given the very small sample size and specialized environment, these findings should be considered preliminary. They are intended to demonstrate capability and generate hypotheses rather than to dictate clinical practice. This proof-of-concept work lays the groundwork for future research with larger cohorts, which will be necessary to confirm these observations and evaluate their clinical significance. The ultimate goal of such research is to translate improved anatomical understanding into strategies that safeguard maternal pelvic health during delivery, but any clinical implications must await further evidence.

## Supplementary information

**Additional file 1:** **Fig. S1:** Anonymized image of the open MRI scanning setup during the SSL.

## Data Availability

The datasets used and/or analyzed during the current study are available from the corresponding author on reasonable request.

## Abbreviations

3D: Three-dimensional
CP: Distance from the coccyx to the sacral promontory
LAM: levator ani muscle
MRI: Magnetic resonance imaging
SCSP: Distance from the inferior edge of the pubic symphysis to the coccyx
SSL: Second stage of labor
TALA: Tendinous arch of levator ani

## Declarations

### Use of Large Language Models (LLMs)

ChatGPT (OpenAI, GPT-5.1, accessed November 2025) was used for assistance with language editing and refinement of the manuscript. No part of the data analysis, results of generation, or scientific interpretation was performed using AI tools. All content was reviewed, corrected, and approved by the human authors, who take full responsibility for the final version.

### Study subjects or cohorts overlap

Some study subjects or cohorts have been previously reported in Maran et al [17], Ami et al [18], and Ami et al [19].

## References

[1] Sherer D, Miodovnik M, Bradley K, Langer O (2002) Intrapartum fetal head position II: comparison between transvaginal digital examination and transabdominal ultrasound assessment during the second stage of labor. Ultrasound Obstet Gynecol 19:264–268. 10.1046/j.1469-0705.2002.00656.x 11896948

[2] Youssef A, Maroni E, Ragusa A (2013) Fetal head-symphysis distance: a simple and reliable ultrasound index of fetal head station in labor. Ultrasound Obstet Gynecol 41:419–424. 10.1002/uog.12335 23124698

[3] Hinkson L, Araujo Junior E, Moron A (2015) Ultrasound during the second stage of labour: is it effective to reduce the caesarean section rates? Rev Bras Ginecol Obstet 37:249–251. 10.1590/SO100-720320150005308 26200821

[4] Dietz H (2004) Ultrasound imaging of the pelvic floor. Part I: two-dimensional aspects. Ultrasound Obstet Gynecol 23:80–92. 10.1002/uog.939 14971006

[5] Zaretsky M, McIntire D, Twickler D (2003) Feasibility of the fetal anatomic and maternal pelvic survey by magnetic resonance imaging at term. Am J Obstet Gynecol 189:997–1001. 10.1067/s0002-9378(03)00835-4 14586343

[6] Bamberg C, Rademacher G, Güttler F et al (2012) Human birth observed in real-time open magnetic resonance imaging. Am J Obstet Gynecol 206:505.e1. 10.1016/j.ajog.2012.01.011 22425409

[7] Lum M, Tsiouris A (2020) MRI safety considerations during pregnancy. Clin Imaging 62:69–75. 10.1016/j.clinimag.2020.02.007 32109683

[8] Dietz HP, Lanzarone V (2005) Measuring engagement of the fetal head: validity and reproducibility of a new ultrasound technique. Ultrasound Obstet Gynecol 25:165–168. 10.1002/uog.1765 15505817

[9] Bamberg C, Scheuermann S, Fotopoulou C (2012) Angle of progression measurements of fetal head at term: a systematic comparison between open magnetic resonance imaging and transperineal ultrasound. Am J Obstet Gynecol 206:161.e1–161.e5. 10.1016/j.ajog.2011.10.867 22177192

[10] Magnin P, Bremond A, Salomon B et al (1975) [Diagram for the prognosis of cephalo-pelvic disproportions. Application in 300 cases of pelvic contraction]. J Gynecol Obstet Biol Reprod 4:975–987 1219054

[11] Caldwell W, Moloy H (1938) Anatomical variations in the female pelvis: their classification and obstetrical significance. Proc R Soc Med 32:1–30 19991699 10.1177/003591573803200101 PMC1997320

[12] Borell U, Fernstrom I (1967) The mechanism of labour. Radiol Clin North Am 5:73–85 6019921

[13] Benavides L, Wu J, Hundley A (2005) The impact of occiput posterior fetal head position on the risk of anal sphincter injury in forceps-assisted vaginal deliveries. Am J Obstet Gynecol 192:1702–1706. 10.1016/j.ajog.2004.11.047 15902181

[14] Nandikanti L, Sammarco A, Kobernik E, DeLancey J (2018) Levator ani defect severity and its association with enlarged hiatus size, levator bowl depth, and prolapse size. Am J Obstet Gynecol 218:537–539. 10.1016/j.ajog.2018.02.005 29454870

[15] Ampt A, Patterson J, Roberts C, Ford J (2015) Obstetric anal sphincter injury rates among primiparous women with different modes of vaginal delivery. Int J Gynecol Obstet 131:260–264. 10.1016/j.ijgo.2015.06.025 26489488

[16] Hehir M, Rubeo Z, Flood K (2017) Anal sphincter injury in vaginal deliveries complicated by shoulder dystocia. Int Urogynecol J. 10.1007/s00192-017-3351-2 28523399

[17] Maran J-C, Cassagnes L, Delmas V et al (2018) Comparative anatomy on 3-D MRI of the urogenital sinus and the periurethral area before and during the second stage of labor during childbirth. Surg Radiol Anat 40:371–380. 10.1007/s00276-017-1925-9 28948372

[18] Ami O, Maran JC, Gabor P et al (2019) Three-dimensional magnetic resonance imaging of fetal head molding and brain shape changes during the second stage of labor. PLoS One 14:e0215721. 10.1371/journal.pone.0215721 PMC6519794 31091263

[19] Ami O, Maran JC, Musset D et al (2022) Human birth imaging using MRI demonstrates fetal head moldability and brain compression: prospective cohort study. JMIR Form Res. 10.2196/27421 PMC9752457 36322921

