# Supplementary material for: MRI visualization of maternal pelvic and perineal anatomical changes during the second stage of labor: a feasibility study
Source: Eur Radiol Exp. 2026 Sep 11;10:129. doi: 10.1186/s41747-026-00757-4 (PMC13569799; doi:10.1186/s41747-026-00757-4)
Supplement: Supplementary file 1 [file 41747_2026_757_MOESM1_ESM.pdf]

MRI visualization of maternal pelvic and perineal anatomical changes during the second stage of labor: A feasibility study

ELECTRONIC SUPPLEMENTARY MATERIAL

Fig. S1: Anonymized image of the open MRI scanning setup during the SSL

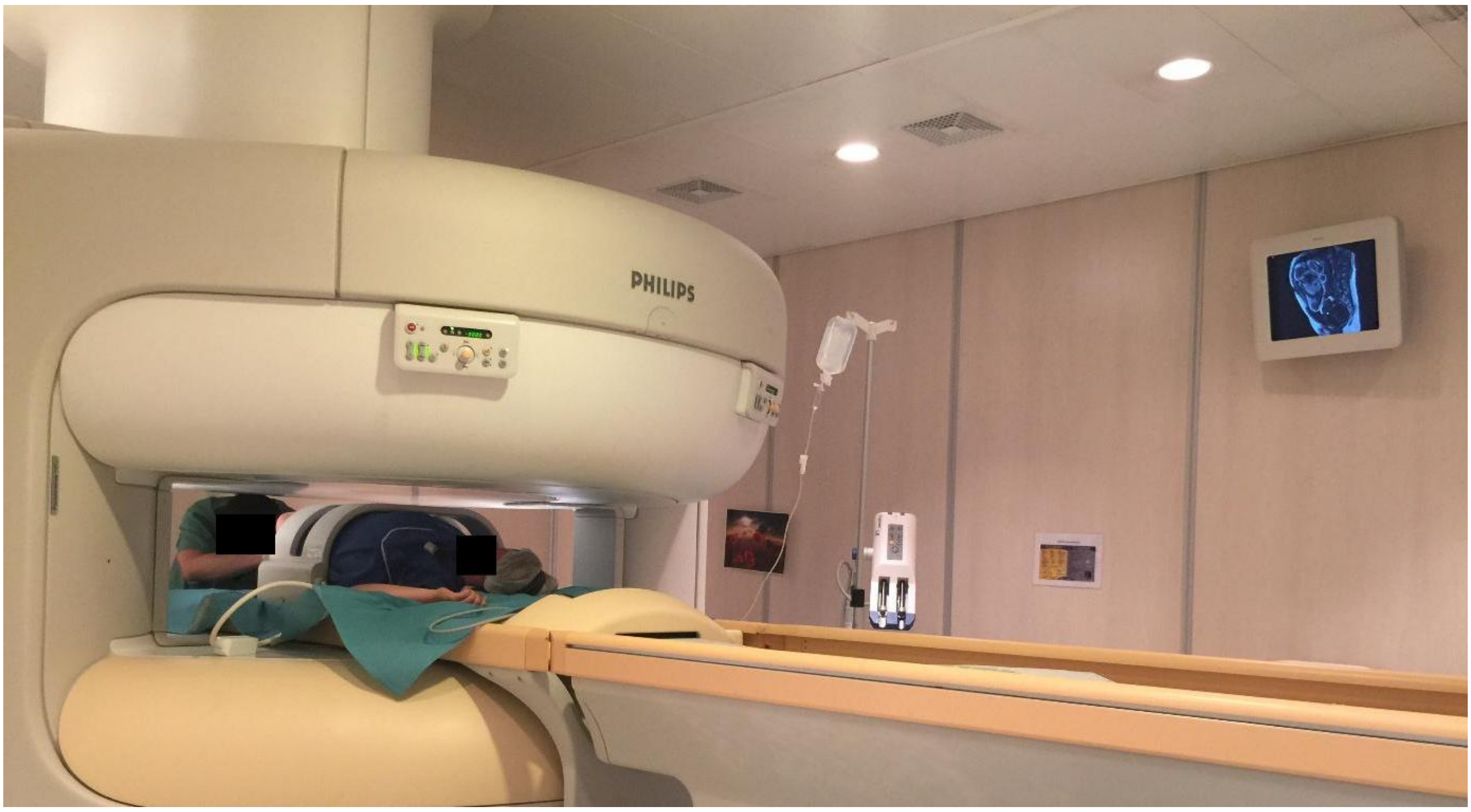

Eur Radiol Exp (2026) Maran JC, Ami O, Dao TT, Mage G, Boyer L.
